# Plant Biostimulants Enhance Tomato Resilience to Salinity Stress: Insights from Two Greek Landraces

**DOI:** 10.3390/plants13101404

**Published:** 2024-05-17

**Authors:** Theodora Ntanasi, Ioannis Karavidas, George P. Spyrou, Evangelos Giannothanasis, Konstantinos A. Aliferis, Costas Saitanis, Vasileios Fotopoulos, Leo Sabatino, Dimitrios Savvas, Georgia Ntatsi

**Affiliations:** 1 Laboratory of Vegetable Production, Department of Crop Science, Agricultural University of Athens, Iera Odos 75, 11855 Athens, Greece; ntanasi@aua.gr (T.N.); karavidas@aua.gr (I.K.); gspyrou@aua.gr (G.P.S.); giannothanasis@aua.gr (E.G.); dsavvas@aua.gr (D.S.); 2 Laboratory of Pesticide Science, Department of Crop Science, Agricultural University of Athens, Iera Odos 75, 11855 Athens, Greece; konstantinos.aliferis@aua.gr; 3 Department of Plant Science, Macdonald Campus, McGill University, Montreal, QC H9X 3V9, Canada; 4 Laboratory of Ecology and Environmental Sciences, Department of Crop Science, Agricultural University of Athens, Iera Odos 75, 11855 Athens, Greece; saitanis@aua.gr; 5 Department of Agricultural Sciences, Biotechnology and Food Science Cyprus University of Technology, P.O. Box 50329, 3603 Lemesos, Cyprus; vassilis.fotopoulos@cut.ac.cy; 6 Department of Agricultural, Food and Forest Sciences, University of Palermo, 90128 Palermo, Italy; leo.sabatino@unipa.it

**Keywords:** sustainability, vegetables, salt stress, tolerance, seaweed extract, microbial

## Abstract

Salinity, one of the major abiotic stresses in plants, significantly hampers germination, photosynthesis, biomass production, nutrient balance, and yield of staple crops. To mitigate the impact of such stress without compromising yield and quality, sustainable agronomic practices are required. Among these practices, seaweed extracts (SWEs) and microbial biostimulants (PGRBs) have emerged as important categories of plant biostimulants (PBs). This research aimed at elucidating the effects on growth, yield, quality, and nutrient status of two Greek tomato landraces (‘Tomataki’ and ‘Thessaloniki’) following treatments with the *Ascophyllum nodosum* seaweed extract ‘Algastar’ and the PGPB ‘Nitrostim’ formulation. Plants were subjected to bi-weekly applications of biostimulants and supplied with two nutrient solutions: 0.5 mM (control) and 30 mM NaCl. The results revealed that the different mode(s) of action of the two PBs impacted the tolerance of the different landraces, since ‘Tomataki’ was benefited only from the SWE application while ‘Thessaloniki’ showed significant increase in fruit numbers and average fruit weight with the application of both PBs at 0.5 and 30 mM NaCl in the root zone. In conclusion, the stress induced by salinity can be mitigated by increasing tomato tolerance through the application of PBs, a sustainable tool for productivity enhancement, which aligns well with the strategy of the European Green Deal.

## 1. Introduction

Salinity detrimentally affects crops through two mechanisms: short-term osmotic stress, resulting in reduced water absorption, and long-term disturbances in ion balance, resulting in ion toxicity phenomena [1,2]. Research findings indicate that salinity affects a substantial portion of agricultural land worldwide [3,4]. Approximately 7% of irrigated areas and up to 33% of the world’s cultivable land experience the detrimental effects of salt accumulation stemming from excessive fertilizer application and crop intensification management practices coupled with irrigation water scarcity [5,6,7]. The estimated annual losses in the agricultural sector that are caused by salinity have been estimated at USD 27.3 billion, thereby positioning this stress as one of the greatest threats to the agricultural sector in the 21st century [8,9,10]. Salinity stress can prevent normal development of horticultural crops, leading to yield losses and fruit size reduction [11]. The accumulation of sodium (Na^+^) and chlorine (Cl^−^) ions in both the plant tissues and the soil is the principal detrimental consequence of salinity [12,13,14]. Furthermore, the presence of NaCl in the root environment can lead to assimilation problems and imbalances of the macronutrients potassium (K), calcium (Ca), and magnesium (Mg) in various plant tissues [15]. Reductions in absorption have also been recorded for the micronutrients copper (Cu), zinc (Zn), iron (Fe), and manganese (Mn), for which reduced transport to the plants’ above-ground parts was also found [16,17,18]. Sustainable, efficient, and economically viable agronomic practices able to mitigate the hazardous effects mentioned above and maintain or even increase yield and food quality are of great significance and must be adapted by farmers [19].

A novel approach for stress mitigation relies on the application of plant biostimulants (PBs) [20,21,22,23,24,25]. PBs are increasingly being incorporated into agricultural systems to adjust physiological functions of plants, thereby enhancing productivity [26,27,28]. While biostimulants do not directly furnish nutrients to plants, they can facilitate nutrient acquisition by supporting metabolic processes within both plants and soil [29]. Various categories of biostimulants exist, encompassing seaweed extracts, protein hydrolysates, humic and fulvic acids, inorganic compounds, beneficial microorganisms, etc. [30,31,32,33,34]. According to Bulgari et al. [35], biostimulants can improve plant tolerance to abiotic stresses, such as drought, extreme temperatures, and salinity, aiding in the recovery from stress-induced damage.

Seaweeds (SWEs) encompassing multicellular macroscopic marine algae from various taxonomic groups like brown (Phaeophyta), red (Rhodophyta), and green (Chlorophyta) algae [36], are considered vital sources of nutrients, fatty acids, polyphenols, proteins [37], bioactive compounds such as laminarins and alginates [38], and phytohormones (cytokinins and auxins) [39]. Utilized in agriculture since ancient times [40], SWE extracts are widely recognized for their dual role in mitigating abiotic stress and enhancing plant productivity [41]. The application of the extracts can be achieved through the foliage or from the soil, thereby increasing the nutrient uptake and the soil chemical properties, respectively [42]. SWEs have been shown to alleviate the effects of salinity stress and elevate potassium (K) and calcium (Ca) concentrations in leaves [43]. Moreover, SWEs contribute to reducing the uptake of sodium (Na) [39,44], while promoting the accumulation of stress-related compounds such as glucosinolates and phytoalexins [45] and antioxidant enzymes such as catalase, superoxide dismutase, and peroxidase [44]. Furthermore, it has been demonstrated that the application of SWE can enhance the quality of fruits, resulting in increased titratable acidity (TA), ascorbic acid, and sugars [46].

Microbial biostimulants, such as *Azotobacter* spp., arbuscular mycorrhizal fungi (AMF), *Rhizobium* spp., and *Azospirillum* spp., constitute another major class of plant biostimulants [47]. These microorganisms, referred to as plant growth-promoting rhizobacteria (PGPRs), can promote plant growth and enhance the ability of plants to withstand abiotic stresses [48] including salinity [49,50]. A recent study demonstrated that the application of PGPB *Azospirillum* brasilense DSM 2298 has the potential to enhance the production and nutritional status of eggplant plants [51]. Conversely, in lettuce plants, the application of different PGPBs (*A. brasilense* DSM 1690, *A. brasilense* DSM 2298 and *Pseudomonas* sp. DSM 25356) was shown to improve the quality and yield of the crop, regardless of the N treatment [52]. This is achieved via an increase in symbiotic nitrogen fixation (SNF) ability, the synthesis of phytohormones, siderophore production, and nutrient uptake. Furthermore, PGPRs facilitate better solubilization of different sources of immobilized phosphorus (P) [53,54] and potassium (K) release from minerals in soils through production of organic acids such as citric, oxalic, tartaric, succinic, and α-ketogluconic acids [55,56,57]. Classified into two categories [extracellular (ePGPR) and intracellular (iPGPR)] [58], PGPRs can stimulate lateral root formation through IAA production (e.g., *Azospirillum brasilense*—[59]) or fix N from the atmosphere (e.g., *Azotobacter vinelandii*—[60]). Azotobacter are considered non-symbiotic N_2_-fixing bacteria and therefore can serve as the primary natural N source in ecosystems lacking SNF [61]. They are highly diverse and globally widespread in soils and can also impact plant growth through cytokinin production [62], P solubilization [63,64], soil carbon and sulfur content increase [65], and stress resilience enhancement (including salinity) [66]. However, the above responses of crops to biostimulants are cultivar-dependent [50,67].

Tomato (*Solanum lycopersium* L.) is the most productive vegetable crop in Southern Europe, covering an extensive area of 0.2 million hectares [68]. Being a moderate salt-stress-resilient crop [69], its productivity can be compromised by high-salinity conditions in the root environment [70,71], mainly ascribed to nutrient and hormonal imbalances, root density inhibition, photosynthesis disruption, and reactive oxygen species (ROS) accumulation [72]. Native to the Pacific shore of South America [73], successfully domesticated in Mediterranean countries (Italy and Spain) and cultivated in marginal lands and under diverse microclimates, tomato landraces are considered a valuable genetic material for increased stress resilience, adaptability to low-input farming systems, and fruit quality [74,75,76]. Recent studies have revealed high economic profitability coupled with increases in sustainability through cultivation of landraces [77], encouraging their exploration, utilization, and even promotion in local food systems as an emerging management strategy [78,79] for agricultural systems with increased resilience and sustainability [80]. Nevertheless, modern varieties frequently demonstrate higher productivity than landraces. One potential sustainable approach to boost yield in landraces involves the application of plant biostimulants (PBs). A number of studies have examined the impact of biostimulants on hybrid and modern cultivars [81]. Nevertheless, research conducted on genotypes [82] has indicated that the application of biostimulants can enhance the nutritional and quality characteristics of genotypes, with the observed variations being linked to the specific type of biostimulant and the genotype under consideration.

Taking all the above into account, a study was designed aiming to evaluate the impact of two commercial biostimulants, an *Ascophyllum nodosum* extract called ‘Algastar^®^’ and a microbial biostimulant known as ‘Nitrostim^®^’, on the response of two Greek tomato landraces to moderate salinity stress induced via a 30 mM NaCl concentration in the root zone. Through identifying possible differential responses among the tested landraces and the different biostimulants under both control and stress conditions, guidelines for assisting tomato growers to achieve higher yields and optimal fruit quality characteristics can be developed.

## 2. Results

### 2.1. Yield Parameters

**Figure 1:**
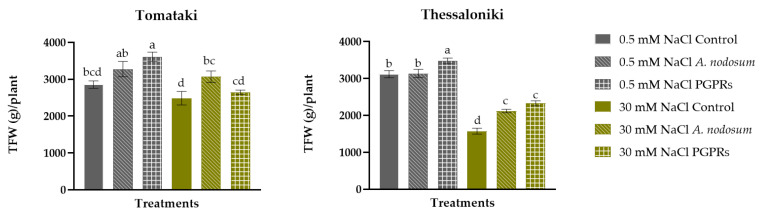
Impact of moderate salinity stress (30 mM NaCl) and plant biostimulants (PBs) on total fruit weight per plant (TFW) of the two Greek tomato landraces, ‘Tomataki’ (small-sized fruit size) and ‘Thessaloniki’ (mid-type fruit size). For each treatment, different letters at each bar indicate significant differences according to Duncan’s multiple range test (*p* < 0.05). Vertical bars indicate standard errors of means (*n* = 4).

Under salinity stress, both cultivars showed a decline in fresh fruit weight per plant (Figure 1). A significant interaction among the two factors (salinity × biostimulant) was observed for both landraces. Specifically, PGPR application proved most effective in enhancing fruit yield of plants cultivated under control conditions, with increases of 20% for ‘Tomataki’ and 12% for ‘Thessaloniki’, respectively, surpassing the untreated plants subjected to 0.5 mM NaCl in the nutrient solution. For Tomataki, under salinity stress conditions, only the application of *A. nodosum* resulted in enhanced yield (23% compared with the untreated control). Regarding ‘Thessaloniki’, both biostimulants significantly increased yield under the stress applied, compared with the untreated salinized plants (an increase of 34% for *A. nodosum* and 48% for PGPR, respectively). In the ‘Tomataki’ landrace plants, the decrease in yield under salinity stress was primarily due to a decrease in mean fruit weight rather than a reduction in fruit numbers (Table 1). Remarkably, the use of biostimulants in ‘Tomataki’ increased the number of fruits compared with the untreated tomato plants. Specifically, the application of *A. nodosum* to ‘Tomataki’ landrace subjected to salt stress effectively maintained the average fruit weight at levels similar to those of the control. Regarding the mid-type cultivar ‘Thessaloniki’, the decline in yield of plants subjected to moderate saline conditions can be attributed to a reduction in both fruit numbers and average fruit weight. Notably, for the stress applied, fruit numbers decreased by 34%, accompanied by a 7% decrease in the mean fruit weight compared with the control. However, the application of the different biostimulants had a beneficial effect on both fruit numbers and mean weight compared with those of the untreated plants. It is worth noting that both biostimulants managed to maintain the fruit weight of the stressed plants to levels comparable to those of the control.

Moderate salinity stress significantly reduced the diameter of both distinct fruit-type Greek landraces (Table 1). In specific, the diameter of the ‘Tomataki’ landrace decreased by 9% under 30 mM of NaCl in the NS, while for the mid-type cultivar ‘Thessaloniki’, the decrease was 5%, both compared with the respective control (0.5 mM NaCl). Biostimulants influenced the diameter of only the small-sized ‘Τomataki’ landrace. Specifically, the application of both *A. nodosum* and PGPRs significantly increased the fruit diameter of stressed plants compared with the untreated control under the same NaCl levels in the NS. Notably, bi-weekly foliar application of *A. nodosum* managed to maintain the fruit diameter of the stressed plants at levels comparable to those of the control. However, the mid-type cultivar ‘Thessaloniki’ did not exhibit any significant differences in fruit diameter associated with biostimulant application nor any interaction between the treatments applied (salinity × biostimulant).

### 2.2. Fruit Quality

Under saline stress conditions, both TSSC and TA levels significantly increased in terms of fruit quality (Table 2). The landrace ‘Tomataki’ showed a 6% rise in TSSC and a 15% increase in TA, while ‘Thessaloniki’ demonstrated a more pronounced increase of 12% in TSSC and 21% in TA. However, only the mid-type landrace experienced a decrease in fruit firmness under stress conditions. Moreover, no discernible benefits in relation to the quality characteristics recorded in this study were observed for this landrace following the application of biostimulants. On the other hand, statistical analysis revealed a strong correlation between salinity and biostimulants for the ‘Tomataki’ landrace. Specifically, when plants were grown under control conditions, the use of *A. nodosum* resulted in notably higher levels of FF compared with the other treatments. Moreover, when treated with this biostimulant, the tomato fruits of plants grown with 0.5 mM NaCl achieved Brix values comparable to those of plants subjected to saline conditions.

### 2.3. Macro- and Micronutrients in Leaves

As shown in Table 3, the salinity factor had a significant effect on all macronutrients in the leaves of both landraces, with the exception of Ca and Mg concentrations in the ‘Tomataki’ landrace. On the other hand, both biostimulants showed a significant effect on only Na concentration in the leaves of the ‘Tomataki’ landrace, while biostimulants seemed to have no effect on Mg concentration in the leaves of the ‘Thessaloniki’ landrace. Moreover, the interaction between salinity and biostimulant factors significantly influenced Na concentration in the leaves of both cultivars. Under salinity stress, Κ concentration decreased by 23% in the leaves of the ‘Tomataki’ landrace and by 21% in ‘Thessaloniki’. However, the effect of the biostimulants for these two parameters was significant only for ‘Thessaloniki’, where the highest concentration in the leaves was found in plants supplied with PBPRs. No interaction with K was found among the tested parameters (salinity stress × biostimulant) for the landrace ‘Tomataki’. A significant increase in Na concentration was observed under stress conditions, approximately sixfold in ‘Tomataki’ and ninefold in ‘Thessaloniki’. The biostimulant factor played a significant role in Na concentration in the leaves of both landraces. Furthermore, the interaction between salinity and biostimulants showed a statistically significant difference between the two landraces. Notably, under stress conditions, the application of both biostimulants reduced leaf Na concentration in ‘Tomataki’, whereas in ‘Thessaloniki’, the application of PGPRs resulted in higher Na accumulation compared with the other treatments. For ‘Tomataki’, Ca and Mg remained unaffected by the two factors studied, as did their interaction. However, for ‘Thessaloniki’, under saline stress conditions, Ca and Mg increased by 38% and 36%, respectively. For this landrace, the application of *A. nodosum* or PGPRs had a significant effect on the concentration of leaf Ca, resulting in higher concentration.

The micronutrients that were assessed in the leaves of the two landraces were Fe and Mn (Table 3). Fe levels decreased significantly by 26% in ‘Tomataki’ subjected to moderate salinity stress conditions, while increased by approximately 29% in ‘Thessaloniki’ under the same conditions. Mn levels decreased by 42% following stress, but only in the ‘Tomataki’ landrace. The biostimulant factor did not have a statistically significant effect on either micronutrient in either landrace. The interaction between salinity and biostimulants showed a statistically significant difference only in leaf Fe concentration of ‘Thessaloniki’. Specifically, bi-weekly foliar application of PGPRs significantly increased Fe concentration in the unstressed plants compared with the untreated control, reaching concentration levels similar to plants subjected to moderate salinity stress.

### 2.4. Macro- and Micronutrients in Fruit

Table 4 shows the concentrations of macronutrients in the fruits. It is clear that the interaction between salinity and biostimulants did not result in any statistically significant differences in any of the macronutrients. It is worth noting that under saline stress, there was a significant tripling of Na concentration in tomato fruits of both genotypes. Additionally, the ‘Tomataki’ landrace exhibited a substantial 20% decrease in Ca concentration in its fruits. The biostimulant factor was found to be statistically significant only for ‘Thessaloniki’. Indeed, for this landrace, the application of *A. nodosum* resulted in the highest concentration of fruit Na. In terms of Mg, PBPRs showed the highest values.

Of the two micronutrients measured in the fruits of both ‘Tomataki’ and ‘Thessaloniki’ cultivars, only Fe exhibited a statistically significant decrease under high NaCl levels in the root zone, with reductions of 19% and 17%, respectively (Table 4). The effect of salinity and the biostimulant on Fe was significant, especially in the case of ‘Tomataki’. The use of *A. nodosum* ensured that the concentration of Fe in the fruits remained constant, regardless of the treatment. Additionally, the application of biostimulants resulted in an increase in Mn concentration in the fruits of ‘Thessaloniki’.

### 2.5. PCA (Principal Component Analysis)

**Figure 2:**
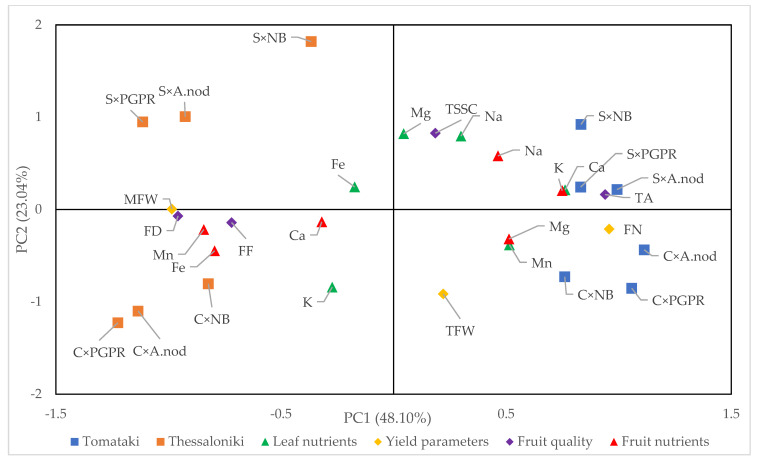
Scores and loading plots were generated to analyze the response of two Greek tomato landraces to the application of plant biostimulants (PBs) in the presence of moderate salinity stress induced under a 30 mM NaCl concentration in the root zone, in terms of yield parameters (TFW, FN and MFW), quality parameters (FD, TSSC, TA and FF), leaf nutrient concentrations (K, Na, Ca, Mg, Fe, and Mn), and fruit nutrient concentrations (K, Na, Ca, Mg, Fe, and Mn). C denotes control conditions (0.5 mM NaCl in the root zone), S denotes stress conditions (30 mM NaCl in the root zone), NB denotes no biostimulant application, A.nod denotes *A. nodosum* application, and PGPR denotes microbial biostimulant application.

Figure 2 presents the principal component analyses (PCAs) including all the parameters and the experimental factors (salinity × variety × biofertilizers) under study. The analysis revealed that the first four principal components (PCs) explained 91.56% of the total variance. PC1, PC2, PC3, and PC4 accounted for 48.10%, 23.04%, 14.60%, and 5.82% of the total variance, respectively. PC1 showed positive correlations with yield parameters (except MFW), fruit TSSC and TA, leaf nutrient profile (except K and Fe), and fruit nutrient content of K, Mg, and Na. Similarly, PC2 was positively collated with MFW, fruit TSSC and TA, leaf nutrient profile (except K and Mg), and fruit nutrient content of Na and K. The two tomato landraces exhibited separation along PC1 and distinct clustering along PC2 in response to salinity levels. Thus, both landraces and salinity significantly contributed to PCA clustering along PC1 and PC2, while the biostimulant treatments did not. Specifically, in Figure 2, the ‘Tomataki’ landrace is positioned in the right quadrants, whereas the ‘Thessoliniki’ landrace occupies the left quadrants. Furthermore, in addition to the noted clustering, plants under salinity stress are represented in the upper quadrants, while those grown under non-saline conditions are positioned in the lower quadrants and under control conditions in the lower right quadrant, irrespective of biostimulant application. Non-saline treatment in plants of the ‘Tomataki’ landrace showed correlations with yield and fruit number per plant, Mn concentration in leaves, and Mg concentration in fruits. Conversely, saline-treated plants were associated with fruit TSSC and TA, leaf content of Na, Ca, and Mg, and fruit content of Na and K. Similarly, non-saline treatment in plants of the ‘Thessaloniki’ landrace correlated with fruit diameter and firmness, K content in leaves, and fruit content of Ca, Mn, and Fe. In contrast, exposure of plants to salinity was correlated only with Fe concentration in leaves.

## 3. Discussion

Tomato plants exposed to high salinity experience physiological disturbances that lead to reduced growth and yield [83]. However, biostimulants have been shown to improve plant resistance and ameliorate the negative impact imposed by abiotic stresses on growth, production, nutritional status, and overall quality [47,84]. In this study, both tomato cultivars exhibited a decrease in fruit weight per plant under saline conditions. The small-sized landrace ‘Tomataki’ had half the yield loss compared with the mid-type fruit landrace ‘Thessaloniki’. This distinction can be attributed to varietal tolerance, which is influenced by fruit morphology in terms of size. This finding aligns well with the findings of the study by Caro et al. [85] who showed that cherry-type tomato cultivars are more tolerant to saline stress than those with normal-sized fruits. Our study showed that the above-mentioned decline in yield of both landraces subjected to salinity stress was influenced by different factors. Specifically, in the small-sized landrace ‘Tomataki’, the reduction of yield under salinity stress appears to have stemmed primarily from a decrease in mean fruit weight, while for ‘Thessaloniki’ it seems to be associated with both a decrease in fruit number and mean fruit weight. According to Eltez et al. [86], yield reduction of in greenhouse hydroponic tomatoes subjected to salinity stress was solely attributed to reduced fruit size. However, in that study, the size of tomato ranged from 57.6–103.1 g per fruit, which was in between the size of the landraces in this study. Consistent with findings of our study, Psarras et al. [87] reported that salinity reduced both the size and number of fruits in Moneymaker, a mid-type cultivar. All the above findings clearly point to a genotype-dependent characteristic of tomato response to salinity stress [78]. The aforementioned decline in fruit numbers can be attributed to physiological alterations induced through the consequent osmotic stress, along with the resultant nutrient imbalance triggered by the elevated salinity in the root zone environment [88].

In this study, the use of biostimulants resulted in a significantly increased yield for both landraces. In the case of ‘Tomataki’, a yield increase of 17% was achieved with the use of PGPRs, and 19% with the foliar application of *A. nodosum*. For ‘Thessaloniki’, the respective increases were 24% and 12%, revealing the genotype–biostimulant specificity [50,67]. According to Ali et al. [89], a 0.5% foliar application of an *A. nodosum* extract enhanced tomato yield by 54%. Similarly, Subramaniyan et al. [90] recorded a 67.8% yield increase when 5.0 L ha^−1^ of *A. nodosum* was applied as soil drenching. Yield increase following *A. nodosum* application, also recorded for other horticultural crops such as sweet pepper [91], pea [92], and watermelon [93], can be attributed to increased assimilation of N use [94] and the stimulation of endogenous hormone homeostasis through the presence of polysaccharides [95]. PGPRs have also been proven to increase tomato yield. According to Katsenios et al. [96] this increase was 51.94% and 45.87% with the application of *Bacillus licheniformis* and *B. subtilis*, respectively. The SNF ability of the *Azotobacter* and *Azospirillum* can impact yield through either nutrient supplementation or through the production of phytohormones such as auxins, cytokinins, and gibberellins [61,62,97]. In the present study, the yield increases in tomato landraces cultivated under control conditions with biostimulant application was either the result of an increase in fruit numbers (‘Tomataki’) or enhancement of both fruit numbers and mean fruit weight (‘Thessaloniki’). According to Ida Di Mola et al. [98], the use of *A. nodosum* extract and a microbial biostimulant containing *Trichoderma afroharzianum* resulted in increased yield of the tomato cultivar ‘Heinz 5108 F1’ (a small-sized type—45 g fruit^−1^) as a result of both higher numbers of fruits and increased mean fruit weight compared with the control. All the above further support genotype–biostimulant specificity. Comparing the effects of biostimulants, the higher yield increase under these conditions was ascribed to PGRP application. The reverse was the case, however, under stress conditions. Indeed, in the present study, ‘Thessaloniki’ increased yield through the application of *A. nodosum* under stress conditions compared with the untreated control and PGPRs, demonstrating its potential in mitigating the adverse environmental impact on crop yield. Moreover, the application of *A. nodosum* resulted in a significant increase in mean fruit weight and fruit diameter of the salinized plants, reaching the control levels. This discovery is consistent with the findings of Di Stasio et al. [99] and can be ascribed to the reduced accumulation of toxic ions such as Na under salt stress. In ‘Tomataki’ however, both biostimulants showed similar yield increase under the stress applied (7% and 23%, respectively). This can be attributed to the increased stress resilience of this tomato cultivar type under salinity stress [78].

Meza et al. [100] attributed the positive impact of salinity stress on fruit TSSC of traditional tomato varieties to osmotic adjustments that help plants in maintaining water uptake levels under stress. Moreover, Agius et al. [101] and Ntanasi et al. [78] concluded that salinity stress can also increase titratable acidity (TA). In this study, both cultivars displayed increased Brix and TA, with the most notable elevation observed in the mid-type ‘Thessaloniki’. The upsurge in TA amidst salinity stress is linked to decreased levels of counterbalancing cations [101]. The significant rise in TSSC and TA observed in the mid-type ‘Thessaloniki’ landrace can also be attributed to the fruit size. According to Caro et al. [85], larger tomato fruits appear to have lower resilience to salt stress compared to small-sized. Furthermore, the presence of 30 mM NaCl in the root zone reduced the firmness of the mid-type cultivar ‘Thessaloniki’ [102,103]. This phenomenon may be attributed to loss of skin elasticity due to reduced cell wall extensibility associated with saline conditions, resulting in reduced resistance to cuticle tearing and cracking, as suggested by Miriam SR et al. [104].

Considering the impact of biostimulants on fruit quality attributes, it was evident that in the cherry-type landrace ‘Tomataki’, the application of *A. nodosum* extract yielded positive effects on both TSSC and fruit firmness (FF). Similarly, a study reported by Ali et al. [89] demonstrated a significant increase in TSSC (°Brix) and FF upon foliar application of *A. nodosum* extract on tomato plants, compared with the control. Previous studies have shown that seaweed extracts, specifically *A. nodosum*, can enhance the ability of tomato plants to withstand environmental stressors such as salinity [105] through inducing various biochemical, physiological, and molecular mechanisms. This, in turn, affects fruit quality parameters, such as acidity. In the present study, TA was increased only in ‘Tomataki’ and remained unaffected in ‘Thessaloniki’. However, the reverse was the case in the studies reported by Ali et al. [89] and Subramaniyan et al. [90], where TA was reduced following *A. nodosum* application. This suggests that the effectiveness of biostimulants depends on the particular variety/genotype/landrace.

Under saline conditions, alterations in leaf mineral content are primarily attributed to the competitive interactions between sodium ions (Na^+^) and potassium ions (K^+^) [106]. In this study, the addition of NaCl to the nutrient solution resulted in a significant increase in Na concentration in the leaves of both tomato cultivars, reaching levels approximately seven times higher than the control. The significant accumulation of Na in tomato leaves, as opposed to fruits, under high NaCl concentrations, is supported by previous findings [107]. As a result of the salinity stress conditions, both cultivars showed a significant reduction in K concentration in their leaves. Under stress conditions, the landrace ‘Tomataki’ showed a decrease in Fe and Mn concentrations, whereas saline stress led to the accumulation of Ca and Fe in the leaves of ‘Thessaloniki’. Nouck et al. [16] noted variations in salinity tolerance and micronutrient accumulation among different tomato cultivars. Furthermore, the tomato fruits of both cultivars exhibited threefold increases in Na concentrations compared with the control. This observation aligns with Hasegawa et al.’s [108] findings that Na accumulation varies across different parts of the plant, which is also supported by the present study. Significantly, the Fe concentration in the fruit of stressed plants of both varieties was reduced.

In terms of Na accumulation in plant tissues, a variation in the impact of the biostimulant application between the two tomato landraces was noted. In the small-sized landrace ‘Tomataki’, both *A. nodosum* (Algastar) and PGPRs (Nitrostim) resulted in reduced Na concentrations in the leaves compared with the control (13% and 23%, respectively). According to the study reported by Jung et al. [109], an elevated concentration of sodium ions (Na) has an antagonistic effect on potassium (K) ions. This is aligned with the findings of our study, where for the mid-type cultivar ‘Thessaloniki’, the microbial biostimulant notably increased Na and reduced K concentrations in the leaves. It is well documented that commercial *A. nodosum* extracts contain essential nutrients (e.g., nitrogen, phosphorous, potassium, calcium, iron, magnesium, zinc, sodium, and sulfur) [110]. Kumari et al. [111] and Zodape et al. [112] also found improved nutrient content in tomatoes treated with seaweed extracts. In the present study, the algal extracts appear to have enhanced the adaptive mechanism of the treated plants exposed to moderate salinity stress, through promoting increased K accumulation with a simultaneous Na cellular influx reduction [105]. Moreover, both biostimulants caused increases in leaf Ca concentration. It is noteworthy that in ‘Thessaloniki’, PGPRs elevated fruit Na concentration, while both biostimulants also significantly increased Mn concentration in ‘Thessaloniki’ fruits.

## 4. Materials and Methods

### 4.1. Plant Material

To evaluate the ability of the studied biostimulants to alleviate osmotic stress factors, two Greek tomato landraces with different salinity resilience were used in this study. The ‘Tomataki’ landrace, a small-sized variety, served as a salinity-tolerant genotype [78], whereas ‘Thessaloniki’, a mid-sized type landrace, was selected as a salt-sensitive genotype [113]. On 25 February 2022, tomato seeds underwent disinfection through a 20-min shaking process with 15% *v*/*w* Na_3_PO_4_, followed by a thorough rinse with water. Subsequently, the seeds were pre-germinated at 25 °C for a duration of 3 days. On 28 February 2022, the germinated seeds were placed in seed trays filled with a turf–perlite substrate.

### 4.2. Growth Conditions

The greenhouse facilities of the Laboratory of Vegetable Production, Agricultural University of Athens, Greece, were used to host the experiment, which was initiated on 12 April 2022, when the tomato plants (4th–5th true leaf stage) were transplanted into perlite bags (Geoflor Hydro, Perlite Hellas S.A., Volos, Greece). An open hydroponic cultivation system was used as a means of crop production. Salinity stress was induced immediately after transplanting. For each variety, half of the plants were subjected to a moderate salinity stress (30 mM NaCl in the nutrient solution), while the remaining plants served as control and were provided with a solution of 0.5 mM NaCl. The stress level was set at 30 mmol L^−1^, based on findings from Adams (2002) [114] and Sonneveld and Voogt (2009) [115], indicating that tomato plants grown hydroponically can withstand NaCl concentrations of up to about 20 mmol L^−1^ in the root environment without affecting yield. Thus, a salinity level of 30 mmol L^−1^ was applied as a salt stress treatment for the tomato plants. Higher concentrations of NaCl are not advisable as they are seldom encountered in commercial practice. Each treatment consisted of four replications, i.e., four perlite bags per treatment. Each bag accommodated three plants of the same landrace, which were supplied with nutrient solution (NS) from a feeding tank via a pump utilizing a drip irrigation system. Throughout the growing season, the average temperature was sustained at 21 °C during the day and 17 °C at night, facilitated by a heating system.

### 4.3. Nutrient Solution Formula

The soilless NutriSense decision support system, accessible at https://nutrisense.online (accessed on 22 April 2024) [53] was used to prepare the NS for every developmental stage. Concentrated fertilizer solutions were prepared and subsequently diluted at a ratio of 1:100 to form the NS distributed to the plants. Perlite bags were saturated with the starter solution one day before transplanting. These bags were immersed in a starter nutrient solution (see Table 5 for starter solution) for a 24 h period. Following this, the bottom of each bag was cut to facilitate proper drainage of excess NS. Half of the bags were filled with the starter NS with a 30 mM NaCl concentration (salinity treatment). The remaining half were filled with the starter NS containing 0.5 mM NaCl, mirroring the composition of the irrigation water (control treatment). Post-transplantation, both control and salt-treated plants received regular watering with an NS (vegetative solution), maintaining a concentration of 30 mM in the rhizosphere for stressed plants and 0.5 μM for control plants, as described in Ntanasi et al. [78]. Throughout the experiment, in addition to the starter NS, three other distinct NSs were employed, tailored to specific growth stages (one for the vegetative and two for the reproductive growth stage, respectively). The NS pH was adjusted daily to 5.6 using a 1 N HNO_3_ solution. Na concentration in the drainage solution was monitored weekly to regulate the NaCl level [78]. A detailed presentation of macronutrient and micronutrient concentrations for each treatment and growth stage is provided in Table 5.

### 4.4. Application of Biostimulants

Two biostimulants were applied one week after transplanting and every 15 days thereafter (see Table S1). The first biostimulant, marketed under the trade name ‘Algastar^®^’ (Mugavero fertilizers, Termini Imerese, Italy) [116] was a seaweed extract of *Ascophyllum nodosum* containing 1% total organic nitrogen (N), 10% total organic carbon (C), and 30% total organic substance, with nominal molecular weight <50 kDa. The second biostimulant, marketed under the trade name ‘Nitrostim^®^’ (Humofert S.A., Athens, Greece) [117], is a microbial solution containing endophytic nitrogen-fixing bacteria at a population of 1 × 10^12^ cfu L^−1^, where cfu stands for colony forming unit, and containing 0.1% manganese (Mn). To prepare the biostimulant solutions, ‘Algastar^®^’ and ‘Nitrostim^®^’ were diluted in 2 mL/L and 5 mL/L of water, respectively. The biostimulants were then applied to the foliage, flowers, and fruits, with full coverage. A nylon-type plastic cover was inserted between the plants to prevent wetting of neighboring tomato plants.

### 4.5. Total Yield and Yield Components

The initial harvest occurred on 1 June, approximately 1.5 months following the transplanting of the crops in the greenhouse. The fruits were collected at their commercial maturity stage. This harvesting routine was replicated roughly three times per week until the termination of the experiment. The recorded parameters encompassed the overall number of fruits per plant, their total fresh weight (g plant^−1^), and the mean fresh weight (g).

### 4.6. Quality Traits

To assess the organoleptic characteristics of the fruits in each sampling, fruits from all varieties and treatments were carefully selected, and their color was measured. Quality parameters such as fruit diameter, fruit firmness, fruit acidity (FA), and total soluble solids content (TSSC °Brix) were meticulously recorded from fruits exhibiting the same color and deemed marketable [118]. Approximately ten fruits per treatment were subjected to these measurements. Fruit diameter was determined using an electronic digital caliper (micrometer accuracy 0.05–150 mm). Fruit firmness was gauged using a mechanical force meter (Chatillon penetrometer—DPP5KG, CHA, St Catharines, ON, Canada). For fruit acidity (FA), 10 mL of tomato juice was subjected to potentiometric titration with 0.02 M NaOH at pH 8.1. A refractometer (Schmidt & Haensch HR32B, Berlin, Germany) was used to record data on TSSC°.

### 4.7. Leaf and Fruit Sampling for Nutrient Analysis

At the time of harvest, fruit samples were collected from all treatments. When the experiment was terminated, leaf samples (from the 3rd, 4th, and 5th leaf) were collected from each plant (4 replications per treatment). The collected plant material was placed in an oven and dried at 65 °C until a consistent weight was achieved. Subsequently, the dried plant materials were ground using a MF 10 Basic Micro Fine Grinder (IKA Werke, Staufen, Germany). For nutrient analyses, dry ashing was applied as an extraction method. Potassium (K) and sodium (Na) in the resulting extract were recorded with the use of a flame photometer (Sherwood Model 410, Cambridge, UK). Calcium (Ca), magnesium (Mg), zinc (Zn), iron (Fe), copper (Cu), and manganese (Mn) were determined with the application of an atomic absorption spectrophotometer (AA-7000, Shimadzu Co., Ltd., Tokyo, Japan).

### 4.8. Statistical Analysis

In the present study, two-way ANOVA analysis was used to identify the main effects of NaCl stress on marketable yield, quality traits, and plant tissue nutrient composition with the different types of biostimulants. Statistical evaluation of the data was performed through ANOVA using the STATISTICA software package, version 12.0 for Windows. For each treatment, 4 replicates of 3 plants each were planned, resulting in a total of 144 plants arranged in a randomized complete block design (RBCD). After the analysis of variance, the means of all evaluated parameters (salinity and biostimulants) were compared for each landrace separately, using Duncan’s multiple range test (*p* ≤ 5%).

## 5. Conclusions

This study demonstrates that different tomato cultivars, with varying fruit sizes, responded differently to salt stress. Salinity generally reduced yield but enhanced fruit quality attributes such as TSSC (°Brix) and TA. However, it is important to note the positive effects of biostimulants. Specifically, the application of ‘Algastar’ was shown to increase fruit diameter and TSSC (°Brix), while ‘Nitrostim’ treatment was linked to improved fruit firmness and higher Fe concentration of salinity-stressed plants. These findings, supported by literature review, not only highlight the significant role of biostimulants in mitigating saline stress but also indicate that biostimulants should serve as strategic tools to optimize the productivity of the crops under adverse growth conditions. However, their efficiency relies on various factors, including cultivar, fruit characteristics, and the specific formulation of the biostimulant used. Therefore, further research and field trials are necessary to determine the optimal application methods to customize biostimulant use to the unique requirements of different tomato landraces.

## Figures and Tables

**Table 1:** Impact of moderate salinity stress (30 mM NaCl) and plant biostimulants (PBs) on fruit numbers per plant (FN), mean fruit weight (MFW), and fruit diameter (FD) of the two Greek landraces, ‘Tomataki’ (small-sized fruit size) and ‘Thessaloniki’ (mid-type fruit size).

		Greek Landraces					
		‘Tomataki’			‘Thessaloniki’		
Salinity Stress	PB	FN (no plant^−1^)	MFW (g)	FD (mm)	FN (no plant^−1^)	MFW (g)	FD (mm)
Main effects (Salinity stress)							
0.5 mM NaCl		69.36	46.91 a	54.41 a	19.69 a	165.12 a	77.43 a
30 mM NaCl		67.14	40.75 b	49.43 b	13.00 b	152.75 b	73.86 b
Main effects (Biostimulant)							
	Control	62.25 b	43.02	50.39 b	15.38 b	149.67 b	75.46
	*A. nodosum*	70.25 a	45.14	52.72 a	16.13 ab	162.13 a	75.15
	PGPRs	72.25 a	43.33	52.28 ab	17.54 a	165.01 a	76.75
Interaction							
0.5 mM NaCl	Control	61.08	46.93 a	53.30 ab	19.00	164.46 a	79.10
	*A. nodosum*	72.17	45.40 a	53.95 ab	19.75	159.24 a	76.37
	PGPRs	74.83	48.41 a	56.05 a	20.33	171.66 a	77.25
30 mM NaCl	Control	63.42	39.11 b	47.28 d	11.75	134.87 b	71.52
	*A. nodosum*	68.33	44.89 a	51.63 bc	12.50	165.01 a	73.45
	PGPRs	69.67	38.25 b	49.13 cd	14.75	158.36 a	76.25
Significance							
Salinity Stress		NS	***	***	***	**	**
PB		*	NS	*	*	*	NS
Salinity Stress × PB		NS	*	*	NS	**	NS

Mean values (*n* = 4) for yield parameters and (*n* = 10) for diameter labeled with different letters within the same column indicate significant differences, as determined through Duncan’s multiple range test (*p* ≤ 5%). Regarding the variance components, according to associated F values, *** (*p* ≤ 0.001), ** (*p* ≤ 0.01), and * (*p* ≤ 0.05) represent statistical significance, while NS signifies non-significance.

**Table 2:** Impact of moderate salinity stress (30 mM NaCl) and plant biostimulants (PBs) on total soluble solids content (TSSC), titratable acidity (TA), and fruit firmness (FF) of two Greek landraces, ‘Tomataki’ (small-sized fruit size) and ‘Thessaloniki’ (mid-type fruit size).

		Greek Landraces					
		‘Tomataki’			‘Thessaloniki’		
Salinity Stress	PB	TSSC (°Brix)	TA (g Citric Acid per 100 g Juice)	FF (kg/cm^2^)	TSSC (°Brix)	TA (g Citric Acid per 100 g Juice)	FF (kg/cm^2^)
Main effects (salinity stress)							
0.5 mM NaCl		4.60 b	0.59 b	1.02	4.45 b	0.34 b	1.36 a
30 mM NaCl		4.89 a	0.69 a	1.02	5.00 a	0.41 a	1.18 b
Main effects (biostimulant)							
	Control	4.58 b	0.64	1.01 b	5.06 a	0.40	1.32
	*A. nodosum*	4.92 a	0.66	1.10 a	4.68 b	0.36	1.29
	PGPRs	4.67 b	0.62	0.96 b	4.51 b	0.39	1.23
Interaction							
0.5 mM NaCl	Control	4.23 c	0.59	0.94 c	4.69	0.35	1.36
	*A. nodosum*	4.91 ab	0.60	1.26 a	4.49	0.34	1.38
	PGPRs	4.60 b	0.59	0.91 c	4.21	0.33	1.35
30 mM NaCl	Control	5.00 a	0.68	1.09 b	5.37	0.45	1.27
	*A. nodosum*	4.93 ab	0.76	0.97 c	4.81	0.37	1.18
	PGPRs	4.76 ab	0.64	1.00 bc	4.82	0.42	1.12
Significance							
Salinity Stress		**	***	NS	***	***	***
PB		*	NS	***	**	NS	NS
Salinity Stress × PB		**	NS	***	NS	NS	NS

Mean values (*n* = 10) labeled with different letters within the same column indicate significant differences, as determined using Duncan’s multiple range test (*p* ≤ 5%). Regarding the variance components, according to associated F values, *** (*p* ≤ 0.001), ** (*p* ≤ 0.01), and * (*p* ≤ 0.05) represent statistical significance, while NS signifies non-significance.

**Table 3:** Impact of moderate salinity stress (30 mM NaCl) and plant biostimulants (PBs) on the concentration (mg/g dry weight (DW)) of macronutrients (K, Na, Ca, and Mg) (mg/g dry weight (DW)) and micronutrients (Fe and Mn) (μg/g dry weight (DW)) in the leaves of two Greek landraces, ‘Tomataki’ (small-sized fruit size) and ‘Thessaloniki’ (mid-type fruit size).

		Greek Landraces											
		‘Tomataki’						‘Thessaloniki’					
Salinity Stress	PB	K	Na	Ca	Mg	Fe	Mn	K	Na	Ca	Mg	Fe	Mn
Main effects (salinity stress)													
0.5 mM NaCl		29.00 a	1.35 b	36.52	5.21	62.65 a	238.14 a	30.50 a	0.54 b	22.06	4.44 b	51.26 b	142.67
30 mM NaCl		22.33 b	7.97 a	33.41	5.03	46.12 b	137.33 b	24.17 b	5.39 a	30.53	6.05 a	66.33 a	150.55
Main effects (biostimulant)													
	Control	24.75	5.30 a	34.39	5.16	54.61	165.80	26.50 b	3.21 a	24.37 b	5.45	56.40	146.66
	*A. nodosum*	25.75	4.60 b	36.31	5.19	51.44	195.11	28.87 a	2.50 b	28.72 a	5.12	57.58	144.42
	PGPRs	26.50	4.08 b	33.92	5.01	56.09	193.25	26.63 b	3.19 a	26.41 ab	5.13	62.11	148.20
Interaction													
0.5 mM NaCl	Control	29.75	1.30 d	38.57	4.97	65.58	239.51	30.25 a	0.52 c	19.05	4.56	44.86 c	133.22
	*A. nodosum*	27.75	1.30 d	35.38	5.29	63.56	237.16	30.50 a	0.55 c	25.59	4.10	49.06 bc	145.67
	PGPRs	29.50	1.45 d	36.13	5.32	59.56	238.10	30.75 a	0.56 c	22.42	4.57	59.32 ab	149.87
30 mM NaCl	Control	19.75	9.30 a	31.26	5.31	46.39	110.53	22.75 c	5.90 a	29.69	6.34	67.95 a	160.11
	*A. nodosum*	23.75	7.90 b	37.24	5.08	39.33	153.06	27.25 b	4.45 b	31.85	6.14	66.09 a	143.17
	PGPRs	23.50	6.70 c	31.72	4.69	52.63	148.40	22.50 c	5.83 a	30.39	5.68	64.91 a	146.53
Significance													
Salinity Stress		***	***	NS	NS	***	***	***	***	***	***	***	NS
PB		NS	***	NS	NS	NS	NS	*	***	*	NS	NS	NS
Salinity Stress × PB		NS	***	NS	NS	NS	NS	*	***	NS	NS	*	NS

Mean values (*n* = 4) labeled with different letters within the same column indicate significant differences, as determined with Duncan’s multiple range test (*p* ≤ 5%). Regarding the variance components, according to associated F values, *** (*p* ≤ 0.001), and * (*p* ≤ 0.05) represent statistical significance, while NS signifies non-significance.

**Table 4:** Impact of moderate salinity stress (30 mM NaCl) and plant biostimulants (PB) on the concentration (mg/g dry weight (DW)) of macronutrients (K, Na, Ca, and Mg) (mg/g dry weight (DW)) and micronutrients (Fe and Mn) (μg/g dry weight (DW)) in the fruit of two Greek landraces, ‘Tomataki’ (small-sized fruit size) and ‘Thessaloniki’ (mid-type fruit size).

		Greek Landraces											
		‘Tomataki’						‘Thessaloniki’					
Salinity Stress	Biostimulant	K	Na	Ca	Mg	Fe	Mn	K	Na	Ca	Mg	Fe	Mn
Main effects (salinity stress)													
0.5 mM NaCl		39.83	0.44 b	0.15 a	1.12	26.81 a	10.56	34.07	0.31 b	0.15	1.03	35.79 a	12.08
30 mM NaCl		43.08	1.48 a	0.12 b	1.10	21.83 b	10.12	36.57	0.76 a	0.15	1.02	29.70 b	11.77
Main effects (biostimulant)													
	Control	41.75	0.95	0.13	1.09	24.74	10.26	34.33	0.53 ab	0.16	0.96 b	32.38	10.76 b
	*A. nodosum*	41.25	0.97	0.15	1.13	23.13	10.51	36.25	0.63 a	0.15	1.05 ab	33.03	12.91 a
	PGPRs	41.38	0.97	0.13	1.11	25.10	10.24	35.88	0.46 b	0.14	1.10 a	32.04	12.54 a
Interaction													
0.5 mM NaCl	Control	39.00	0.43	0.15	1.08	29.66 a	10.92	33.67	0.28	0.14	0.96	34.53	11.18
	*A. nodosum*	37.25	0.46	0.16	1.12	23.40 bc	10.43	34.50	0.41	0.15	1.05	37.71	12.52
	PGPRs	43.25	0.45	0.14	1.15	27.39 ab	10.33	34.25	0.28	0.14	1.12	35.78	12.99
30 mM NaCl	Control	44.50	1.47	0.10	1.10	19.82 c	9.61	35.00	0.79	0.16	0.97	30.77	10.34
	*A. nodosum*	45.25	1.48	0.13	1.13	22.86 bc	10.60	38.00	0.85	0.14	1.04	28.36	13.22
	PGPRs	39.50	1.50	0.12	1.07	22.81 bc	10.15	37.50	0.65	0.15	1.08	29.05	12.10
Significance													
Salinity Stress		NS	***	***	NS	**	NS	NS	***	NS	NS	**	NS
PB		NS	NS	NS	NS	NS	NS	NS	**	NS	*	NS	**
Salinity Stress × PB		NS	NS	NS	NS	*	NS	NS	NS	NS	NS	NS	NS

Mean values (*n* = 4) labeled with different letters within the same column indicate significant differences, as determined via Duncan’s multiple range test (*p* ≤ 5%). Regarding the variance components, according to associated F values, *** (*p* ≤ 0.001), ** (*p* ≤ 0.01), and * (*p* ≤ 0.05) represent statistical significance, while NS signifies non-significance.

**Table 5:** Nutrient concentrations in the different nutrient solutions supplied to tomato plants. Starter solution: (0 days after transplanting (DAT); vegetative stage, from 2nd DAT to 35th DAT (blossom of 3rd truss); reproductive stage 1, from 36th DAT to 48th DAT (blossom of 3rd to 5th truss); reproductive stage 2, from 48th DAT (blossom of 5th truss up to the termination of crop).

Nutrient	Starter Solution (11 April 2022)	Vegetative Stage (13 April 2022)	Reproductive Stage 1 (19 May 2022)	Reproductive Stage 2 (1 June 2022)	Unit
NO^3−^	16.76	14.70	14.04	14.79	mM
K^+^	7.50	8.88	8.53	8.70	mM
Ca^2+^	9.40	5.36	5.39	5.49	mM
Mg^2+^	4.25	2.51	2.34	2.38	mM
SO_4_^2−^	7.19	3.59	3.80	3.42	mM
H_2_PO_4_^−^	1.20	1.42	1.45	1.45	mM
NH4^+^	1.34	1.28	1.91	1.46	mM
Fe	15.00	20.00	19.19	19.19	μM
Mn^++^	10.00	10.00	9.86	9.86	μM
Zn^++^	5.00	6.50	6.54	6.54	μM
B	30.00	35.00	36.93	36.93	μM
Cu^++^	0.75	0.80	1.00	1.00	μM
Mo	0.50	0.50	0.90	0.90	μΜ
Cl^−^	4.00	2.80	3.00	3.00	μΜ

## Data Availability

Data are presented in the paper. Raw data can be provided upon request.

## Supplementary Materials

The following supporting information can be downloaded at: https://www.mdpi.com/article/10.3390/plants13101404/s1, Table S1: The biostimulants used in tomato cultivation and the dates of application.
