# Supplementary material for: Plant Biostimulants Enhance Tomato Resilience to Salinity Stress: Insights from Two Greek Landraces
Source: Plants (Basel). 2024 May 17;13(10):1404. doi: 10.3390/plants13101404 (PMC11125247; doi:10.3390/plants13101404)
Supplement: Supplementary file 1 [file plants-13-01404-s001.zip › plants-3001264-supplementary.pdf]

Table S1: The application dates of the biostimulant are listed alongside the respective product.

Application dates	Biostimulants	
19 April 2022	Algastar	Nitrostim
27 April 2022	Algastar	-
29 April 2022	-	Nitrostim
17 May 2022	Algastar	Nitrostim
27 May 2022	Algastar	Nitrostim
15 June 2022	Algastar	Nitrostim
1 July 2022	Algastar	Nitrostim
